# HeartMate 3 Explantation Using Felt Plug for Ventriculotomy Occlusion

**DOI:** 10.14797/mdcvj.1208

**Published:** 2023-05-08

**Authors:** Melissa Medina, Amit Alam, Amarinder Bindra, Nishi Patel, Cesar Guerrero-Miranda, Katharina Fetten, Dan M. Meyer, Aldo E. Rafael-Yarihuaman

**Affiliations:** 1 Baylor Scott & White Heart and Vascular Institute, Baylor University Medical Center, Dallas, Texas, US; 2 Annette C. and Harold C. Simmons Transplant Institute, Baylor Scott & White Research Institute, Dallas, Texas, US; 3 Center for Advanced Heart and Lung Disease, Baylor University Medical Center, Dallas, Texas, US; 4 Texas A&M Health Science Center, Baylor University Medical Center, Dallas, Texas, US; 5 WellSpan Health, York Hospital, York, Pennsylvania, US

**Keywords:** LVAD explantation, myocardial recovery, ventriculotomy occlusion

## Abstract

Left ventricular assist devices (LVAD) can be utilized for heart failure patients as a bridge to transplant, bridge to destination, or bridge to recovery. Given the lack of a universally accepted consensus for assessing myocardial recovery, techniques and strategies in LVAD explantation also vary. In addition, the incidence of LVAD explantation remains relatively low, and surgical techniques of explantation continue to be areas of interest. Our approach using a felt-plug Dacron technique is an effective way to preserve left ventricular geometry and cardiac function.

## Introduction

We describe a technically reproducible approach for ventriculotomy closure during HeartMate3 (HM3; Abbott Cardiovascular) left ventricular assist device (LVAD) explantation. A 35-year-old Hispanic male with a past medical history of polysubstance abuse (methamphetamine, marijuana, and cocaine), presented with cardiogenic shock SCAI stage D, INTERMACS 1 profile. He was not responding to dual inotropic therapy with escalating requirements of milrinone and dobutamine. The decision was made to implant an HM3 LVAD. At the time of HM3 implant, pathology of the left ventricular apical core specimen showed a marked increase in subepicardial adipose tissue and focal contraction-band necrosis most consistent with idiopathic dilated cardiomyopathy. His postoperative course was unremarkable.

After initiation of guideline-directed medical therapy and abstinence from further drug use, he presented to the emergency room 9 months later with asymptomatic low flow alarms of < 1 L/min at 5500 rotations per minute (RPM). Transthoracic echocardiogram (TTE) showed a well-seated HM3 device and inflow cannula, a left ventricular ejection fraction (LVEF) of 40% to 45%, a 4.6-cm LV end-diastolic diameter, and notable opening of the aortic valve with each beat. There were no suction events, arrythmias, nor evidence of hemolysis or pump thrombosis (lactate dehydrogenase 213 U/L). There was no evidence of end-organ damage with normal creatinine (0.78 mg/dL) and liver function tests (bilirubin 0.6 mg/dL, AST 24 U/L, ALT 52 U/L); international normalized ratio was therapeutic (2.8), and the patient had New York Heart Association class 1 complaints. A ramp study was performed, slowly decreasing the speed from 5500 RPM to 4000 RPM, with peak exercise to 8.6 metabolic equivalents being achieved (Table 1). LV recovery was concluded, and he was deemed a candidate for explantation.

**Table 1:** Criteria for left ventricular assist device explantation. RPM: rotations per minute; LPM: liters per minute; PI: pulsatility index; LVEF: left ventricular ejection fraction; LVEDD: left ventricular end-diastolic diameter; MR: mitral regurgitation; AV: aortic valve; PA: pulmonary artery; PCWP: pulmonary capillary wedge pressure; CO: cardiac output; CI: cardiac index; Sat: saturation


RPM	FLOW (LPM)	POWER (WATTS)	PI	LV EF	LV EDD	MR SEVERITY	AV	PA (MM HG)	PCWP (MM HG)	CO/CI (L/MIN/M^2^)	PA SAT

5500	4	6.7	4.5	40–50%	4.6	mild	opens	29/11	9	6.2/2.8	71%

5000	2.8	4.4	6.5	40–50%	4.7	mild	opens	29/9	12	5.8/2.6	70%

4500	0.9	2.9	10	40–50%	4.7	mild	opens	32/16	13	5.7/2.6	67%

4000	1.5	2.9	8.8	40–50%	4.7	mild	opens	63/27	23	13/5.9	67%


## Surgical Technique and Procedure

**Figure 1:**
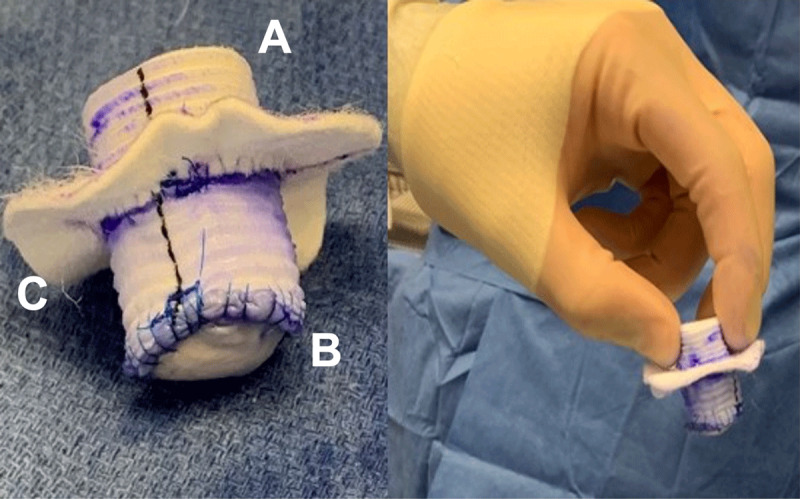
Felt Dacron plug creation. **(A)** Rolled PTFE felt placed within 20-mm Hemashield graft. **(B)** 20-mm circular pericardial patch was sewn onto one end of the graft. **(C)** 1.5-mm PTFE felt ring with 20-mm central circular opening was sewn to the tubular graft, 1 cm away from the pericardial patch end. A second identical felt ring was attached to the first ring using bio-glue as a sealant. PTFE: polytetrafluoroethylene

**Figure 2:**
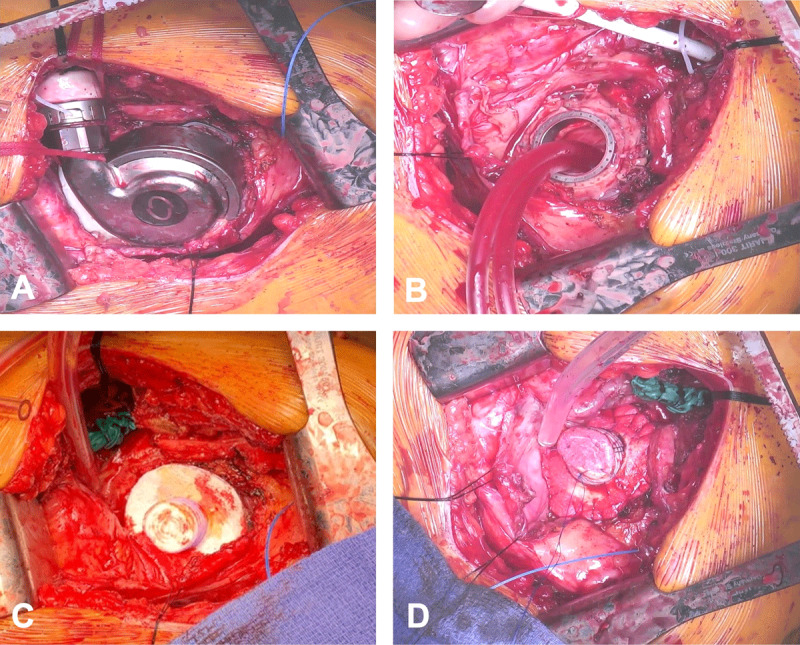
Implanted custom-made skirted synthetic plug. **(A)** HeartMate3 pump, bend relief and driveline exposed. **(B)** While on cardiopulmonary bypass, device turned off, outflow graft stapled, driveline cut, and pump removed. **(C, D)** Custom-made skirted synthetic plug inserted into the ventricular core and fixed onto the sewing ring.

A left anterolateral thoracotomy at the sixth interspace was performed. LV function was assessed without cardiopulmonary bypass and the LVAD off, and the patient continued to be appropriate for explantation. Peripheral cannulation was done and cardiopulmonary bypass commenced. A plug was made using a rolled polytetrafluoroethylene (PTFE) felt, which was sewn into a 20-mm collagen-impregnated Hemashield Platinum woven double velour polyester graft (Maquet, Getinge Group). Next, a 20-mm pericardial patch was sewn to one end of the tubular graft using 4-0 prolene. A 20-mm circular opening was made within a PTFE felt ring, approximately 15 mm in width, and sewn into the tubular graft 1 cm away from the pericardial patch end (Figure 1). A second identical felt ring was attached to the first ring using bio-glue as a sealant. The HM3 pump, bend relief, and driveline were exposed. On cardiopulmonary bypass, the device was turned off, the outflow graft stapled, the driveline cut, and the pump removed. The previously described skirted synthetic plug was inserted into the ventriculotomy core and fixed onto the sewing ring using 3-0 prolene (Figure 2). The patient was separated from cardiopulmonary bypass successfully with stable hemodynamics.

**Figure 3:**
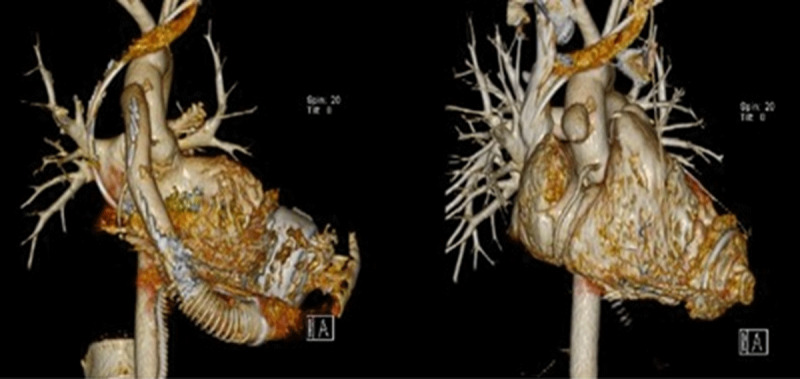
Pre- and postoperative computed tomography scans. Residual left ventricular assist device fixation ring present with hemostatic synthetic plug and notable absence of left ventricular thrombus.

A postoperative computed tomography (CT) scan demonstrated the residual LVAD fixation ring with the hemostatic synthetic plug present and without any notable LV thrombus (Figure 3). The patient had an uneventful hospital course; systemic heparin was initiated on postoperative day 2, and the patient was discharged on postoperative day 6 with an LVEF of 50% on TTE. Anticoagulation was continued for 6 months after explantation without any notable sequelae. Guideline-directed medical therapy ensued postoperatively. A TTE obtained 15 months after explant showed that the LV apical function appeared recovered. LV systolic function was moderately decreased, EF was 35% to 39%, and there was grade I mild diastolic dysfunction. The LV cavity was normal in size, with a 5.3-cm LV internal dimension, moderate global hypokinesis, and no regional contraction abnormalities. These echocardiography findings were also similarly unchanged at 2 years post-explant. Currently, there is no consensus on anticoagulation post-LVAD explantation. Since LV wall motion, including the apex, appeared preserved, we decided not to anticoagulate beyond 6 months.

## Discussion

LVAD explantation following successful myocardial recovery is a rare entity, with literature citing 1% to 2% occurrence.^1^,^2^ While the pathophysiology and myocardial recovery mechanisms are largely understudied, there is not a universally accepted standardized criteria specific for HM3 LVAD explantation. Reports from various centers use cardiac reserve evaluation^3^ and scoring prediction models such as INTERMACS Cardiac Recovery Score (I-CARS)^2^ in addition to LVAD-weaning strategies utilizing multidisciplinary teams, right heart catheterization, laboratory and echocardiography data, and intraoperative assessments.^4^,^5^,^6^ For our patient—with stable vitals and end-organ parameters, hemodynamic stability seen on TTE and right heart catheterization after decreasing LVAD speeds, and an I-CARS score of 8—our multidisciplinary team felt comfortable with the decision to proceed with LVAD explantation.

An additional factor to consider is the high perioperative morbidity and mortality risk for patients undergoing LVAD explantation due to prior adhesions, bleeding, and infection risks. It is prudent to keep operative and cardiopulmonary bypass times short, and therefore minimally invasive approaches are also being attempted to minimize operative times and reduce the aforementioned risks. Several approaches such as ligation of the outflow tract, device and sewing ring removal with subsequent ventriculoplasty, or occlusion of the sewing ring have been described in literature.^4^,^7^,^8^,^9^,^10^,^11^,^12^

Surgical approaches described for LVAD explant include redo-sternotomies, left anterior thoracotomies, and left subcostal incisions with options to (1) simply ligate the outflow graft, leaving the LVAD in situ; (2) explant the LVAD followed by plication of the left ventriculotomy; or (3) fixate plugs onto the ring. As Baldwin et al. described following a HeartMate2 (HM2) explant, leaving the driveline in place after its transection may pose an infection risk postoperatively.^13^ Moreover, LV plication may distort ventricular geometry or affect LV wall motion and possibly increase operative times. Alternatives have been described, such as using pericardial patches, felt plugs,^13^,^14^,^15^ or custom metal plugs.^7^,^9^,^10^,^11^ Preserving the sewing ring and using plugs that are fixed onto the ring allows for the possibility of LVAD re-implantation should the future need occur.

The ease and practical nature of using a felt-plug Dacron technique is an effective way to preserve LV geometry and cardiac function without the need for increased cardiopulmonary bypass and operative times. We do not have experience with another explant technique specific to the HM3; rather, our approach is based on an HM2 explantation technique previously published by Cohn et al.^14^ Furthermore, with titanium plugs only approved for compassionate use in the United States (and not FDA approved), our Dacron technique is safe, cost-effective, and technically reproducible for ventriculotomy closure that maintains minimal cardiac manipulation intraoperatively and preserves ventricular geometry.

## Future Directions

Our case highlights areas of knowledge and research that warrant further investigation. Firstly, we identified that there is a small percentage of patients who will have LV recovery that requires LVAD explantation, and a need for a standardized approach and criteria is necessary. As such, given the low incidence, any surgical intervention, such as the one described using the felt-plug Dacron technique, may be difficult to study in a randomized fashion when compared with other techniques described in literature. Lastly, a majority of the patients described in literature in whom a plug was used were anticoagulated post-explant for different periods of time due to the potential nidus of thrombus formation as the blood contacts the artificial surface.^7^,^9^,^10^,^16^ Currently, there is no consensus for duration of anticoagulation post-LVAD explantation, and this is currently tailored to each individual patient.

## Conclusion

The ease and practical nature of using a felt-plug Dacron technique is an effective way to preserve LV geometry and cardiac function without the need for increased cardiopulmonary bypass and operative times.
